# Evaluation of Anorexia in Cancer and Its Association with Autonomic Nervous System Activity Assessed by Heart Rate Variability

**DOI:** 10.3390/nu15234936

**Published:** 2023-11-28

**Authors:** Alessio Molfino, Carmen Gallicchio, Giovanni Imbimbo, Michele Melena, Silvia Antonini, Antonietta Gigante, Maurizio Muscaritoli

**Affiliations:** Department of Translational and Precision Medicine, Sapienza University of Rome, 00185 Rome, Italy; carmen.gallicchio@uniroma1.it (C.G.); giovanni.imbimbo@uniroma1.it (G.I.); michele.melena@uniroma1.it (M.M.); silvia.antonini@uniroma1.it (S.A.); antonietta.gigante@uniroma1.it (A.G.); maurizio.muscaritoli@uniroma1.it (M.M.)

**Keywords:** cancer anorexia, autonomic nervous system, malnutrition, body weight loss, heart rate variability

## Abstract

Alterations in the central nervous system in cancer patients are pivotal in determining appetite dysregulation and body weight loss (BWL). Autonomic nervous system activity was tested by measuring heart rate variability (HRV) in cancer patients presenting with anorexia. We considered inpatients with different types of cancer and investigated anorexia using their FAACT scores. HRV was evaluated by a three-channel Holter ECG. The domains of low frequencies (LF, sympathetic activity) and high frequencies (HF, parasympathetic activity) were calculated. Also, SDNN (autonomic activity) and RMSSD (parasympathetic activity) were assessed. We enrolled 56 patients with cancer and 23 controls. In cancer patients, RMSSD and SDNN were lower than in controls (*p* < 0.001 and *p* = 0.009). Sympathetic activity (LF nu) was lower in cancer patients than in controls (*p* = 0.023), including sympathovagal balance (LF/HF nu ratio) (*p* = 0.025). RMSSD was reduced in anorexic (*p* < 0.001) and non-anorexic (*p* = 0.003) cancer patients compared to controls. The SDNN was lower in anorexic cancer patients than in non-anorexic cancer patients (*p* = 0.025), and it was lower in anorexic cancer patients than in controls (*p* = 0.001). LF nu was lower in anorexic cancer patients than in controls (*p* = 0.015), as was LF/HF (*p* = 0.031). SDNN was negatively correlated with BWL in the cancer group (rho = −0.40; *p* = 0.007). Our data support the hypothesis that autonomic nervous system dysregulation exists in patients with cancer presenting with anorexia, with implications for its diagnosis and treatment.

## 1. Introduction

Anorexia is a highly disabling symptom of cancer, being diagnosed in up to 70% of patients with an advanced stage of the disease [1,2,3]. Importantly, poor appetite is crucial in determining impaired food intake and consequent body weight loss [4]. Also, the presence of poor appetite has been associated with lower survival in cancer patients, independent of the presence of body weight loss [5]. Several factors have been indicated to promote the loss of appetite, such as increased circulating levels of proinflammatory cytokines, the presence of neuroinflammation, and changes in plasma tumor-secreted factors [6]. In this light, energy control is mainly regulated in the central nervous system and several evidence, both in animal models and in humans, supported that its alterations are pivotal during cancer [7]. Supporting this hypothesis, by using functional magnetic resonance imaging, we previously demonstrated that cancer patients with anorexia had altered hypothalamic activity compared to those without anorexia [8]. Also, the autonomic nervous system is able to modulate energy homeostasis, and its alterations have been investigated in relation to increased catabolism during cancer [9]. The analysis of heart rate variability (HRV) is an inexpensive, easy-to-use, non-invasive technique evaluating the autonomic modulation of heart rate (HR) through measurements of instantaneous beat-to-beat variations in R-R interval length [10], analyzing the domains of low frequencies (LF, index of the sympathetic modulation) and high frequencies (HF, index of the parasympathetic modulation), as previously described [11].

HRV is considered a useful tool to assess the ability of the heart to respond to physiological and environmental stimuli [11]. It is a marker of the responsiveness of the autonomic nervous system involved in the regulation of vital processes, including blood pressure, respiration, and nutrient digestion. Increasing evidence confirms an association between changes in HRV and mental/psychological and physical health [12]. Greater HRV is correlated with a healthy heart that is able to adapt and respond effectively to stressors, while lower variability indicates a compromised ability to regulate different multi-organ functions [12]. Also, previous studies found that lower HRV was associated with aging, and a positive correlation was observed between HRV and physical activity, as well as with other health-promoting habits [12]. HRV has been used previously to analyze the relationship between nutritional status and altered autonomic activity in humans [13]; however, to our knowledge, no data have been reported specifically regarding the association between anorexia and HRV status in patients with cancer. In light of this, our study aimed at evaluating the changes in autonomic nervous system activity through the analysis of HRV in a group of cancer patients with or without anorexia and in a control group without cancer or anorexia.

## 2. Materials and Methods

### 2.1. Selection of Cancer Patients and Controls

We conducted a cross-sectional, observational, controlled study. We considered subjects who were participating in a study assessing nutritional issues while they were inpatients admitted to the Department of Translational and Precision Medicine, Internal Medicine and Clinical Nutrition Unit, Policlinico Umberto I Hospital, Sapienza University of Rome, Italy. A local Ethics Committee approved the study (protocol number 27/19). All the participants signed an informed consent form and were enrolled from April 2021 to September 2022. Patient inclusion criteria were a confirmed presence of cancer, age ≥ 18 years, and the ability to sign the informed consent form. The exclusion criteria for both groups were the presence of atrial fibrillation, other arrhythmias, or other concomitant highly catabolic disease, the administration of anticancer treatments in the last three months, or the use of beta-blockers. The control group included subjects with normal appetite and without any conditions associated with systemic inflammation, who were followed for hypertension in the same department as the cancer group and treated with diuretics or calcium channel blockers.

### 2.2. Assessment of Anorexia and Nutritional and Inflammatory Parameters

Anorexia was evaluated using the Italian version of the Functional Assessment of Anorexia/Cachexia Therapy (FAACT) score (see Table 1), a questionnaire composed of 12 items scored on a 5-point Likert scale (0 = not at all, 1 = a little bit, 2 = somewhat, 3 = quite a bit, and 4 = very much), with a sum score from 0 to 48, classifying patients as anorexic (FAACT ≤ 30) or non-anorexic (FAACT > 30), as previously described [2,14]. The patients completed the proposed questionnaire with the assistance of medical personnel, if required. To investigate patients’ anthropometric indices, we measured body weight and height, calculated body mass index (BMI), and registered the involuntary loss of body weight in the prior six months. Serum C-reactive protein (PCR) and albumin levels were measured using automated, standard techniques, given their well-known role as biomarkers of inflammation/nutritional status in cancer patients [1].

### 2.3. HRV Assessment

The participants underwent 24 h ambulatory, 3-channel Holter ECG recording (Lifecard CF; Spacelabs Healthcare, Snoqualmie, WA, USA). Autonomic nervous activity was analyzed following the recommendations of the Task Force of the European Society of Cardiology and the North American Society of Pacing and Electrophysiology, as previously described [15]. At 10-min intervals, all the participants were studied with ECG recordings, and the time of registration was split into 2 periods: day (7 a.m. to 12 p.m.) and night (12 p.m. to 7 a.m.). Artificial and arrhythmic data were not considered. In the time domain, we evaluated the standard deviation of normal-to-normal RR intervals (SDNN) (ms) and the mean square root of the differences between adjacent intervals (RMSSD), which are indices of global autonomic activity and parasympathetic activity, respectively [10,15,16]. In the frequency domain, a fast Fourier transform was utilized to obtain power spectral estimates of HRV to document low frequency (LF: 0.04–0.15 Hz, indicating sympathetic activity) and high frequency (HF: 0.15–0.40 Hz, indicating parasympathetic activity) in normalized units (nu). The LF/HF ratio indicates changes in the sympathovagal balance [10,15,16]. The main parameters of HRV analysis are summarized in Table 2.

### 2.4. Statistical Analyses

HRV data analyses were completed using dedicated software (Accuplus 363; Del Mar Avionics, Irvine, CA, USA). The Shapiro–Wilk test was used to assess the normality of the variables. All continuous, normally distributed variables were indicated as means ± SD. Non-normally distributed variables were expressed as medians (IQR). The differences in HRV parameters between anorexic and non-anorexic cancer patients and controls were analyzed using a non-parametric test (Kruskal–Wallis test) and a parametric test (ANOVA), as appropriate. We utilized the two-tailed *t*-test or the Mann–Whitney test according to a normal or non-normal distribution, respectively, to verify differences between groups. Pearson’s or Spearman’s correlations were calculated to test dependence between variables according to a normal or non-normal distribution, respectively. A *p*-value < 0.05 was considered statistically significant. SPSS version 26 was used to perform statistical analyses.

## 3. Results

### 3.1. Participants’ Characteristics

We enrolled a total of 56 cancer patients (26 males, 46%) with a mean age of 70 ± 11 years and a mean BMI of 23.2 ± 4.6 kg/m^2^. Most of the cancer diagnoses were for lung (19/56, 34%), gastrointestinal (10/56, 18%), breast (8/56, 14%), and other cancers (19/56, 34%), including head and neck, liver, brain, urothelial, and blood cancer (Table 3). Cancer patients reported a median body weight loss in the previous six months of 5.6% (0; 12), and 44 patients presented with an advanced stage of the disease (class III–IV) (Table 3).

In cancer patients, the median FAACT score was 32.5 (21.8; 40.0), with a prevalence of anorexia according to FAACT ≤ 30 of 46.4% (26/56) (Table 3).

Cancer patients with cancer anorexia presented with higher C-reactive protein levels than those without anorexia (*p* = 0.005), whereas no differences were found in albumin levels or BMI (Table 3). No differences in terms of CRP and albumin levels were detected according to sex. Anorexic cancer patients tended to have a greater body weight loss in the previous six months compared to non-anorexic cancer patients (*p* = 0.067) (Table 3). Anorexic cancer patients presented with a more advanced stage of cancer disease compared to non-anorexic cancer patients (96% vs. 63%, *p* = 0.003). The main comorbidities reported by cancer patients were cardiovascular disease (17/56, 30%), chronic obstructive pulmonary disease (8/56, 14%), chronic kidney disease (4/56, 7%), and type 2 diabetes mellitus (11/56, 20%) (Table 3). Patients with and without anorexia did not show differences in the prevalence of comorbidities, including diabetes.

The control group included 23 participants (10 males, 38%) recruited in the outpatient clinic at the same study site; control patients had a mean age of 69.1 ± 10 years and a BMI of 25.2 ± 3.5 kg/m^2^, presented with controlled hypertension, and none of them had appetite problems.

### 3.2. Heart Rate Variability in Cancer Patients and Controls

**Figure 1:**
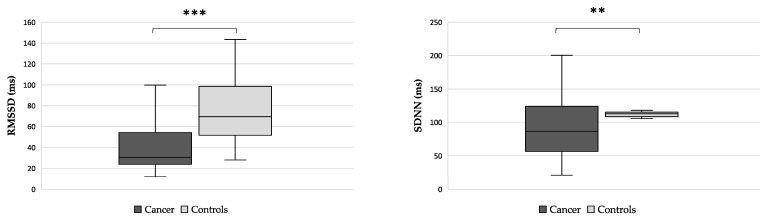
Differences in HRV parameters (RMSSD, left side, and SDNN, right side) between cancer patients and controls. Abbreviations: Mean square root of the differences between adjacent intervals, RMSSD; standard deviation of normal-to-normal RR intervals, SDNN. ** *p* < 0.01, *** *p* < 0.001.

Among cancer patients, the median values of RMSSD and SDNN were lower than among controls (*p* < 0.001 and *p* = 0.009, respectively) (Figure 1).

**Figure 2:**
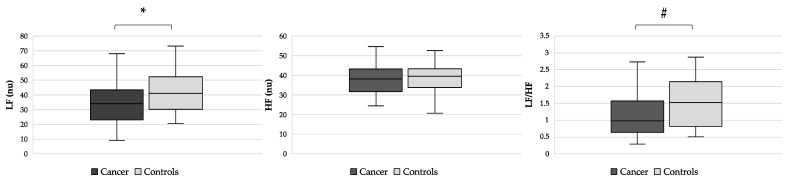
Differences in markers of sympathetic (LF nu, right side), parasympathetic (HF nu, middle), and sympathovagal balance (LF/HF, left side) from HRV analysis between cancer patients and controls. Abbreviations: Low frequency, LF; high frequency, HF; normalized units, nu; heart rate variability, HRV. * *p* = 0.023, # *p* = 0.025.

Also, sympathetic activity, as expressed by LF nu, was reduced in patients with cancer compared with controls (*p* = 0.023) (Figure 2), whereas no difference in parasympathetic activity (levels of HF nu) was detected between the two groups (*p* = 0.846). Sympathovagal balance, evaluated by the LF/HF nu ratio, was lower in cancer patients than in controls (*p* = 0.025) (Figure 2).

### 3.3. Heart Rate Variability between Anorexic and Non-Anorexic Cancer Patients and Controls

**Figure 3:**
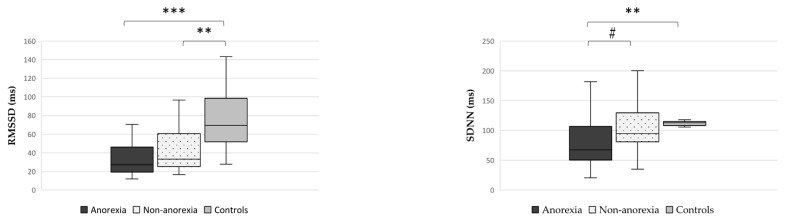
Differences in HRV parameters (RMSSD, left side, and SDNN, right side) between anorexic cancer patients, non-anorexic cancer patients, and controls. Abbreviations: mean square root of the differences between adjacent intervals, RMSSD; standard deviation of normal-to-normal RR intervals, SDNN. # *p* = 0.025, ** *p* < 0.01, *** *p* < 0.001.

Considering the presence of anorexia, RMSSD levels were reduced both in anorexic and non-anorexic cancer patients compared to controls (*p* < 0.001 and *p* = 0.003, respectively) (Figure 3), but no difference was detected between anorexic and non-anorexic cancer patients (*p* = 0.235). Moreover, SDNN levels were lower in cancer patients with anorexia than in those without anorexia (*p* = 0.025), and likewise in anorexic cancer patients compared to controls (*p* = 0.001) (Figure 3), whereas no difference was detected between non-anorexic and controls (*p* = 0.185).

**Figure 4:**
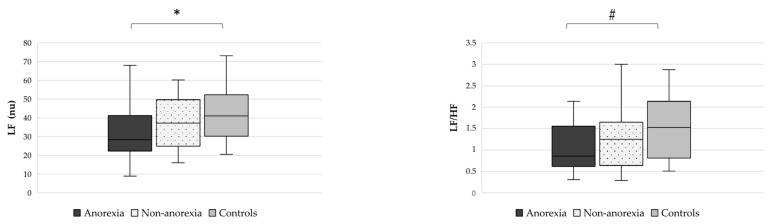
Differences in markers of sympathetic (LF nu, left side) and sympathovagal balance (LF/HF, right side) identified by HRV analysis of anorexic cancer patients, non-anorexic cancer patients, and controls. Abbreviations: Low frequency, LF; high frequency, HF; normalized units, nu; heart rate variability, HRV. * *p* = 0.015, # *p* = 0.031.

Also, LF nu was lower in anorexic cancer patients than in controls (*p* = 0.015) (Figure 4), whereas no differences were observed between anorexic and non-anorexic (*p* = 0.228) cancer patients or between non-anorexic cancer patients and controls (*p* = 0.373). Considering the sympathovagal balance, LF/HF was reduced only in cancer patients with anorexia compared to controls (*p* = 0.031) (Figure 4).

### 3.4. Correlations between HRV Parameters, Anorexia, and Body Weight Loss in Cancer Patients

**Figure 5:**
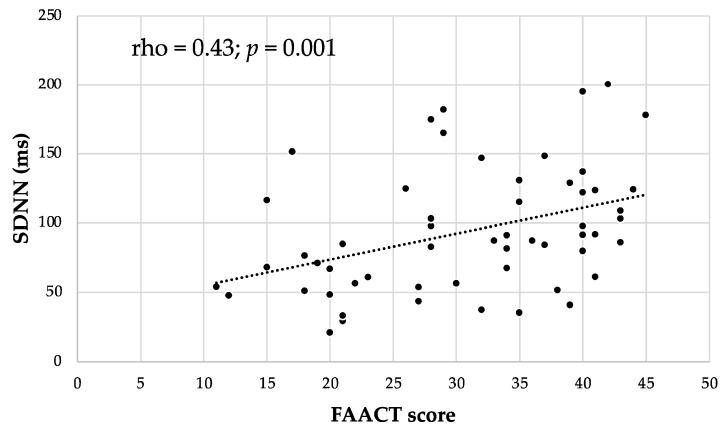
Correlation between SDNN (ms) levels and FAACT score in cancer patients. Abbreviations: standard deviation of normal-to-normal RR intervals, SDNN; Functional Assessment of Anorexia/Cachexia Treatment, FAACT.

In cancer patients, the FAACT score correlated positively with LF/HF (rho = 0.30; *p* = 0.04), LF nu (rho = 0.34; *p* = 0.01), and SDNN (rho = 0.43; *p* = 0.001, Figure 5).

Moreover, SDNN negatively correlated with involuntary body weight loss percentage in the cancer group (rho = −0.40; *p* = 0.007).

## 4. Discussion

In this study, we found differences in parameters of global autonomic activity, as well as in parasympathetic pathways, as assessed by HRV, between cancer patients and controls. In particular, cancer patients presenting with anorexia showed lower values of SDNN and RMSSD when compared to controls. Interestingly, SDNN was also decreased in anorexic cancer patients when compared to non-anorexic cancer patients, but no differences were detected in terms of sympathetic (LF nu) or sympathovagal balance (LF/HF) between anorexic cancer patients and controls.

In our cohort of hospitalized cancer patients, the prevalence of anorexia (observed in more than 40% of the sample) was in accordance with the recent data obtained by studies using the FAACT score as a diagnostic tool [1,18]. Also, cancer patients with impaired appetite presented with higher degrees of inflammation, as indicated by higher C-reactive protein levels, and a trend of increased body weight loss compared to those without anorexia, highlighting the involvement of systemic inflammation in the pathophysiology of anorexia and the impact of poor appetite in determining catabolic status.

Recent data showed the involvement of the autonomic nervous system in the control of the inflammatory response [19]. The main effector of the increased inflammatory status is the vagus nerve, which inhibits the production of pro-inflammatory cytokines through the neurotransmitter acetylcholine [19]. Supporting these hypotheses, Singh et al. [20] showed a negative correlation between some HRV parameters, such as HF and inflammation. The vagal nerve has been shown to produce an inhibitory effect on inflammation, blocking the secretion of tumor necrosis factor-a (TNF-a) by macrophages [20].

Experimental and clinical evidence has indicated that the sympathetic nervous system is able to induce cancer progression and limit the efficacy of anti-cancer treatments [21]. These effects can be attenuated by specific treatments, including, for example, beta-blockers [21]. Also, recent data have shown that specific exercise programs may deliver beneficial effects on autonomic nervous system modulation in cancer patients and survivors [22]. In particular, among cancer survivors, it appears clinically useful to select those patients whose sympathetic imbalance is excessively increased. In keeping with this, some authors found altered cardio-vagal regulation in women with breast cancer and described a higher level of sympathetic modulation shown by specific parameters [17].

Interestingly, in our cohort of cancer patients, the FAACT score was positively correlated with SDNN values, indicating that a preserved appetite correlated well with better autonomic activity. Moreover, SDNN was inversely correlated with involuntary body weight loss, which is one of the most important clinical indicators of poor nutritional status and cachexia in patients with cancer [1].

The involvement of the autonomic nervous system in cancer patients has been described in many studies documenting that cancer patients often suffer from autonomic dysfunction, such as postural hypotension, nocturnal diarrhea, fatigue, bladder-emptying problems, and male erectile dysfunction [23].

Hypothalamic regions involved in autonomic deregulation are mainly represented by the rostral ventrolateral medulla, the nucleus of the solitary tract, and the paraventricular nucleus (PVN), where the NPY neurons involved in appetite control are also located [24]. In fact, Deng X et al. showed that in rats, alterations in sympathovagal balance evaluated by HRV analysis were related to changes in PVN activity. In particular, the authors observed a reduction in LF nu, LF/HF, and SDNN following electric induction of lesions of PVN [25]. This observation is in line with our results documenting a modulation of these HRV parameters when anorexia was present.

The hypothalamus plays a central role in regulating food intake. During cancer, several studies, conducted both in animals and in humans, have described hypothalamic dysfunction associated with anorexia [26]. In particular, the vagus nerve may be sensed by different factors during cancer, including cytokines. Through the transmission of these signals to brainstem regions, the hypothalamus leads to the activation of the melanocortin system through different pathways, promoting catabolic status [26].

The link between inflammation, the autonomic nervous system, and anorexia has been confirmed in tumor-bearing animals. In these experimental models, there was a significant upregulation of hypothalamic IL-1 mRNA expression and elevated levels of IL-1 in the cerebrospinal fluid, which were inversely related to food ingesta [27,28]. Also, in another study, anorexia symptoms were improved by hypothalamic injection of the IL-1 receptor antagonist and by intraperitoneal injection of recombinant human soluble TNF receptor [29]. Regarding the neural control of appetite, POMC/CART neurons located in the arcuate nucleus of the hypothalamus that express type 1 IL-1 receptors play a crucial role in regulating energy balance by suppressing food intake and increasing energy expenditure. Interestingly, intracerebroventricular injection of IL-1 enhanced the frequency of action potentials within the population of POMC/CART neurons [30].

Another important neurotransmitter known to be involved in the development of anorexia in cancer is serotonin [31]. In fact, in animal models, the onset of poor appetite was associated with the elevation of serotonin within the hypothalamus, and intrahypothalamic injection of the serotonin antagonist ameliorated energy homeostasis in anorectic animals [29].

In human subjects, the potential role of serotonin in inducing anorexia has been hypothesized following the observation of increased levels of tryptophan, the precursor of serotonin, in the plasma and cerebrospinal fluid of anorexic and cachectic patients with cancer [32]. In addition, the accumulation of tryptophan in the brain during illness may promote catabolic effects beyond its role as a serotonin precursor [26]. Brain tryptophan is also implicated in the synthesis of kynurenine and its derivatives, which are compounds with immunomodulatory properties [33]. The tryptophan metabolism through the kynurenine pathway is enhanced by inflammation and results in the production of free radical generators. Stress conditions, including cancer, may modulate the levels of both serotonin and tryptophan, with potential implications for HRV that are still unclear and need to be studied in the condition of cancer anorexia.

We previously demonstrated reduced hypothalamic activity in patients with lung cancer, as evaluated by functional magnetic resonance; specifically, we observed a reduced hypothalamic response after oral food challenge in patients with anorexia [8]. In light of these findings, our data confirm the presence of sympathovagal imbalance when developing anorexia, which should be associated with hypothalamic disfunction.

Interestingly, several studies have demonstrated that SDNN represents a prognostic index during cancer disease and other chronic conditions [34,35]. In particular, Kim et al., in a cohort of hospice patients (mainly affected by advanced lung and gastrointestinal cancers), showed that by performing multivariate regression analysis, SDNN and anorexia were both associated with survival [34,36].

In line with previous reports indicating changes in autonomic activity in advanced cancer [23], we showed that LF nu and LF/HF ratio were lower in cancer patients vs. controls, and FAACT score positively correlated with LF nu and LF/HF ratio.

Anorexia represents a key clinical feature in cancer for developing body weight loss and altered nutritional status. In fact, the loss of appetite is known to cause a reduction in calorie and protein intake in many cancer patients. In parallel, cancer patients present with higher energy expenditure, leading to calorie deficiency that can reach more than 1000 kcal/day [37,38].

The development of hypophagia (low food intake), in turn, determines involuntary body weight loss in association with the loss of muscularity and adiposity [7]. In light of this, anorexia and reduced food intake are pivotal in promoting cancer-associated cachexia. In fact, international guidelines have provided indications regarding the minimum energy and protein requirements for cancer patients to avoid the development of body weight loss and malnutrition [39]. In particular, calorie intake should not be less than 25 kcal/kg/day, and a minimum protein intake should be set at 1 g/kg/day [39].

Cancer patients exhibit a globally higher prevalence of malnutrition, ranging from approximately 20% to over 70% according to several variables, such as age, cancer type, and stage [3,39].

Therefore, the data of the present study and those previously obtained indicate that anorexia is common at the time of diagnosis as well as during the cancer journey, highlighting that metabolic and nutritional derangements affect cancer patients with potential negative clinical consequences. The identification of markers of autonomic disfunction may be useful to counteract the development of a severe form of anorexia and body weight loss.

A multimodal strategy aimed at implementing early recognition and treatment of anorexia and consequent inadequate food intake is needed. In view of this, the study of HRV could be easily available to both inpatients and outpatients with cancer with limited costs, and it represents a non-invasive technique.

Our study has some limitations, including the type of participants represented by inpatients with cancer, most of whom were in an advanced stage of the disease. Some cancer patients were diagnosed with diabetes, which is known to affect autonomic nervous system activity; however, no difference in the prevalence of diabetes was found between anorexic and non-anorexic cancer patients. Furthermore, the control group included patients affected by hypertension (outpatients); hypertension was well controlled by therapy in all control patients.

In conclusion, anorexia is a symptom frequently reported by cancer patients in any phase of the disease. Central nervous system dysregulation has been documented and, in our cohort, autonomic dysfunction has been observed according to the presence of cancer and, for some parameters, according to the presence of anorexia. In particular, SDNN levels were decreased when anorexia was present. Interestingly, SDNN was also correlated with involuntary body weight loss, which is a negative clinical consequence of anorexia in cancer. Our results confirm in humans the involvement of the central nervous system in the complex pathophysiology of disease-related anorexia and underline the importance of evaluating this aspect of the diagnosis.

## Figures and Tables

**Table 1:** Functional Assessment of Anorexia/Cachexia Therapy (FAACT) score, adapted from AC/S-12 of the FAACT questionnaire [14].

	Not at All	A Little Bit	Somewhat	Quite a Bit	Very Much
I have a good appetite	0	1	2	3	4
The amount I eat is sufficient to meet my needs	0	1	2	3	4
I am worried about my weight	4	3	2	1	0
Most food tastes unpleasant to me	4	3	2	1	0
I am concerned about how I think I look	4	3	2	1	0
My interest in food drops as soon as I try to eat	4	3	2	1	0
I have difficulty eating rich or heavy food	4	3	2	1	0
My family or friends are pressuring me to eat	4	3	2	1	0
I have been vomiting	4	3	2	1	0
When I eat I seem to get full quickly	4	3	2	1	0
I have pain in my stomach area	4	3	2	1	0
My general health is improving	0	1	2	3	4

**Table 2:** Main parameters used for the heart rate variability (HRV) analysis, adapted from Majerova K et al. [17].

Parameters	Units	Description
**Time domain**		
Mean RR	ms	The mean of RR intervals
SDNN	ms	Standard deviation of RR intervals
Mean HR	min^−1^	The mean heart rate
STD HR	min^−1^	Standard deviation of instantaneous heart rate values
RMSSD	ms	Square root of the mean squared differences between successive RR intervals
pNN50	%	NNxx divided by the total number of RR intervals
HRV triangular index		The integral of the RR interval histogram divided by the height of the histogram
**Frequency domain**		
VLF, LF, and HF peaks	Hz	Peak frequencies for VLF, LF and HF bands
VLF, LF, and HF powers	ms^2^	Absolute powers of VLF, LF and HF bands
VLF, LF, and HF powers	%	Relative powers of VLF, LF and HF bands
LF and HF powers	nu	Powers of LF and HF bands in normalized units: LF [n.u.] = LF [ms^2^]/(total power [ms^2^] − VLF [ms^2^]; HF [n.u.] = HF [ms^2^]/(total power [ms^2^] − VLF [ms^2^])
LF/HF	/	Ratio between LF and HF band powers

Abbreviations: standard deviation of normal-to-normal RR intervals, SDNN; heart rate, HR; standard deviation, STD; the mean square root of the differences between adjacent intervals, RMSSD; very low frequencies, VLF; low frequencies, LF; high frequencies, HF; normalized units, nu; heart rate variability, HRV.

**Table 3:** Patients’ characteristics.

Parameter	Cancer (n = 56)	
	Anorexic (n = 26)	Non-Anorexic (n = 30)
Age, y	67 ± 12	73 ± 10
Males, n (%)	9 (34)	17 (56)
BMI, kg/m^2^	22.5 (19.8; 23.8)	23.1 (20.4; 26.7)
Body weight loss, %	7.6 (1.9; 14.4)	4.5 (0.0; 9.3)
FAACT score ***	22.0 ± 5.6	38.4 ± 3.7
CRP, mcg/L **	71,500 (21,650; 117,350)	14,550 (5225; 30,600)
Albumin, g/dL	3.2 (2.8; 3.6)	3.3 (2.7; 3.6)
*Type of cancer*		
Lung, n (%)	11 (42.3)	8 (26.7)
Gastrointestinal, n (%)	4 (15.4)	6 (20)
Breast, n (%)	4 (15.4)	4 (13.3)
Other, n (%)	7 (26.9)	12 (40)
*Stage of cancer*		
Stage I–II, n (%)	1 (3.8)	11 (36.7)
Stage III–V, n (%) **	25 (96.2)	19 (63.3)
*Main comorbidities*		
COPD, n (%)	4 (15)	4 (40)
Cardiovascular, n (%)	5 (19)	12 (50)
Chronic kidney disease, n (%)	2 (8)	2 (7)
Diabetes, n (%)	6 (23)	5 (17)

Abbreviations. Body mass index, BMI; Functional Assessment of Anorexia/Cachexia Treatment, FAACT; C-reactive protein, CRP; chronic obstructive pulmonary disease, COPD. ** *p* < 0.01, *** *p* < 0.001.

## Data Availability

The data presented in this study are available on request from the corresponding author.
